# Health and personal satisfaction for women at midlife: a middle-range theory

**DOI:** 10.3389/fgwh.2026.1753217

**Published:** 2026-03-18

**Authors:** Rnda I. Ashgar

**Affiliations:** College of Nursing and Health Sciences, Nursing Department, Jazan University, Jazan, Saudi Arabia

**Keywords:** middle-aged, middle-range theory, personal satisfaction, women at midlife, women's health

## Abstract

**Purpose:**

Midlife represents a critical developmental phase for women, characterized by significant physiological, psychological, and social transitions that can profoundly impact their overall well-being. These transitions, such as the menopausal process, shifting family dynamics, and evolving professional roles, can significantly impact a woman's sense of personal satisfaction and contentment, which in turn can substantially impact their overall health and well-being. The current study explores the intricate relationship between personal satisfaction and health outcomes in women at midlife, proposing a middle-range theory to illustrate this connection.

**Methods:**

This middle-range theory was developed using a deductive approach, informed by a nursing conceptual model, namely King's Conceptual System, and a comprehensive review of the relevant literature on the phenomenon.

**Results:**

The synthesis of the literature outlines the impact of various personal satisfaction dimensions on physical and mental health outcomes in women at midlife, which supports the application of the proposed theory as a guiding theory.

**Implications for practice:**

The proposed theory provides valuable insights for healthcare professionals, policymakers, and researchers seeking to promote the health and well-being of women at midlife, addressing the critical need for comprehensive and individualized approaches to healthcare that acknowledge the significance of personal satisfaction as a determinant of overall health.

## 1 Introduction

Midlife represents a pivotal life stage for women, characterized by a confluence of biological, psychological, and social transitions that can significantly impact their overall health and well-being (1, 2). The existing body of research frequently approaches the health of women at midlife through the lens of menopause and reproductive decline, potentially overlooking the significance of subjective experiences, such as personal satisfaction, in shaping their health outcomes (1, 3, 4). Moreover, the existing literature often frames the health of women at midlife in terms of either the presence of problems and ailments or the absence of symptoms and complaints, which may oversimplify the complex interplay of factors influencing their well-being (3, 5). It is imperative to recognize people's increasing life span, which challenges traditional notions of what constitutes midlife, necessitating a more nuanced understanding of this life stage (4). The current paper aims to describe the intricate relationship between personal satisfaction and health in women at midlife, proposing a middle-range theory that elucidates the processes through which satisfaction influences health outcomes during this critical developmental period. By examining the multifaceted nature of personal satisfaction and its connection to various dimensions of health, we seek to provide a more holistic understanding of women's health experiences in midlife.

This manuscript makes several theoretical contributions to the fields of nursing, health, psychology and lifespan development by centering personal satisfaction as a key factor in understanding the health of women at midlife. It expands existing models of subjective well-being through situating personal satisfaction within the unique psychosocial context of midlife, a period often marked by caregiving responsibilities, career transitions, and evolving family roles. Unlike generalized approaches to adult well-being, this manuscript theorizes that personal satisfaction is shaped by midlife experiences, highlighting how midlife presents distinct challenges and opportunities for self-evaluation and emotional fulfillment.

The current study provides valuable insights for healthcare professionals, policymakers, and researchers seeking to promote the health and well-being of women at midlife, addressing the critical need for comprehensive and individualized approaches to healthcare that acknowledge the significance of personal satisfaction as a determinant of overall health.

## 2 Methodology

### 2.1 Theory development

**Figure 1:**
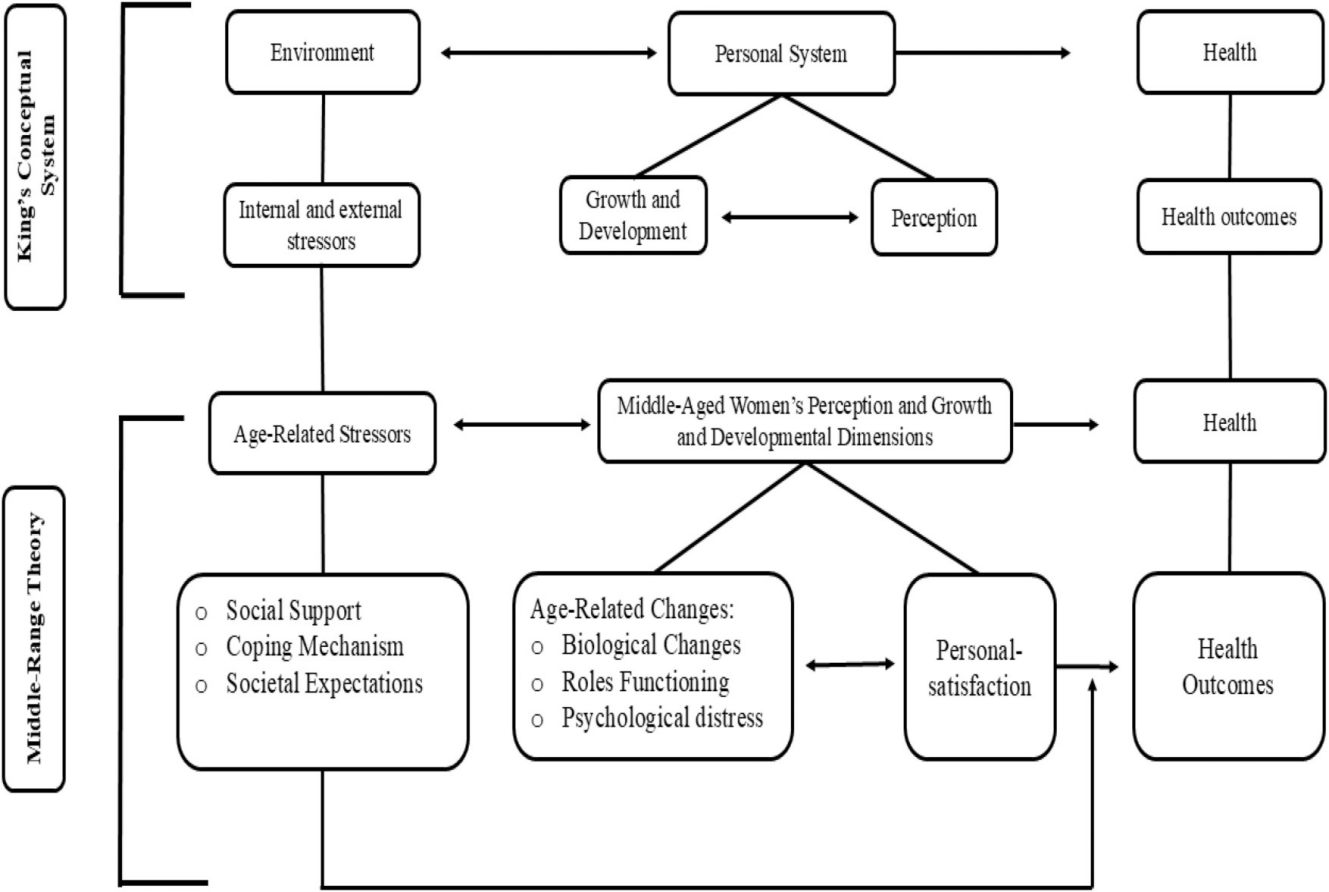
Theory substruction based on king's conceptual system.

This middle-range theory was developed using a deductive approach, informed by a nursing conceptual model and a comprehensive review of the relevant literature on the phenomenon. The conceptual model provided a coherent framework for articulating the relationships among the key concepts of the theory and for outlining its foundational assumptions. Concurrently, the synthesis of literature offered an in-depth understanding of each concept, particularly in the context of the experiences of women at midlife (see Figure 1).

### 2.1.1 Literature review

We employed a scoping review approach, adhering to the methodological framework outlined by the JBI Scoping Review Guidelines (6). This systematic approach ensured a rigorous and comprehensive identification of relevant studies, enabling a thorough exploration of the multifaceted factors influencing middle-aged women's health and satisfaction. The review further delves into how biological changes, evolving role functioning, social support networks, psychological distress, coping mechanisms, and societal expectations collectively shape their well-being and health trajectories.

A systematic search was conducted in major databases, PubMed, PsycINFO, and Web of Science, for peer-reviewed studies published between 2000 and 2025, focusing on women approximately aged 40–65. Search terms included combinations of “life satisfaction,” “personal satisfaction,” “self-satisfaction,” “middle-aged women,” “menopause,” “role strain,” “social support,” “psychological distress,” “coping,” and “health outcomes.” Both quantitative and qualitative studies were included.

**Figure 2:**
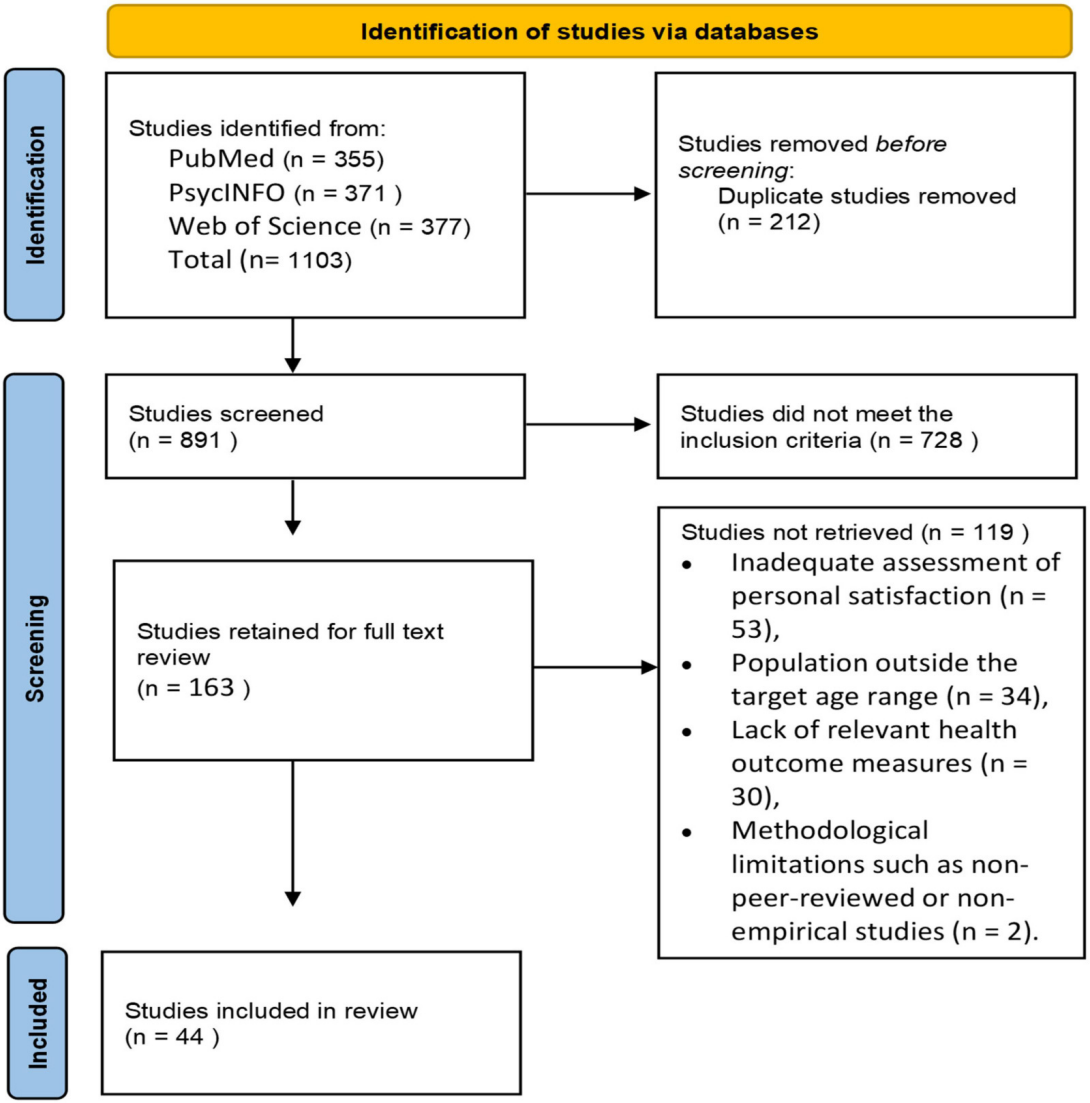
PRISMA flow diagram of the scoping review process.

The initial database search yielded a total of 1,103 articles. After removing 212 duplicates, 891 unique articles remained for screening. Titles and abstracts were assessed for relevance based on predefined inclusion and exclusion criteria. Articles were included if they focused on women aged approximately 40–65 years, examined at least one domain of personal satisfaction, assessed health outcomes or well-being in relation to personal satisfaction or age-related stressors, and were published in English in peer-reviewed journals. Studies were excluded if they focused solely on men or women outside the midlife range, were case reports, conference abstracts, opinion pieces, or did not report original research findings. Following this initial screening, 163 articles were retained for full-text review. During full-text assessment, articles were excluded for reasons including inadequate assessment of personal satisfaction (*n* = 53), population outside the target age range (*n* = 34), lack of relevant health outcome measures (*n* = 30), and methodological limitations such as non-peer-reviewed or non-empirical studies (*n* = 2). After this detailed evaluation, a total of 44 studies met all inclusion criteria and were included in the final scoping review, see Figure 2.

Data were then systematically extracted from included studies using a standardized extraction table developed by the authors. The extracted information included key study variables, methodological features, and findings relevant to the aims of the review. This structured approach was used to ensure consistency, transparency, and reproducibility across the screening and data extraction processes. Insights derived from the scoping review were synthesized to identify recurring patterns and themes, which subsequently informed the selection of the major concepts underpinning the proposed theoretical framework.

### 2.1.2 King's conceptual system

Imogene King developed King's Conceptual System when the nursing profession sought recognition as a legitimate science (7). This system comprises three components: the personal system, the interpersonal system, and the social system (8). The personal system refers to each individual, whether healthy or ill; the interpersonal system pertains to interactions between two or more individuals; and the social system encompasses groups or organizations, such as families or communities (9). The relationships among these three systems are characterized as interactions that reflect the social and physical environments where individuals, families, and communities engage in daily activities to achieve their objectives (9). The primary aim of nursing within King's Conceptual System is to assist individuals, families, groups, and communities in maintaining and restoring health, enabling them to function effectively in their roles (10). A key relational proposition unites all four concepts of the nursing metaparadigm: “The focus of nursing is human beings interacting with their environment leading to a state of health for individuals, which is an ability to function in social roles” (7).

King's Conceptual System has served as a framework to inform various aspects of nursing, including practice, research, administration, and education, addressing multiple areas of focus. Nonetheless, a significant portion of the research grounded in King's Conceptual System tends to concentrate on isolated studies related to specific phenomena (7). Additionally, multiple practice tools and mid-range theories, such as the Theory of Goal Attainment, have been derived directly from King's conceptual framework (7).

## 3 Results

### 3.1 Thematic synthesis

Following data extraction from the scoping review, a thematic synthesis was conducted to identify recurring patterns, domains, and relationships across the included studies. Extracted information on key variables, outcomes, and contextual factors was coded and grouped into preliminary themes. These themes formed the basis for the identification of core constructs and the articulation of relationships within the proposed theoretical framework, see Table 1.

**Table 1:** Summary of scoping review–derived themes and Major theoretical concepts.

Reviewed studies	Scoping review insight	Theme	Major concept in theoretical framework
(11–19)	Higher personal satisfaction is linked to adherence to treatment plans, proactive self-care, regular physical activity, balanced diet, and screening adherence; acts as a protective factor against chronic disease	Positive correlation with preventive health behaviors and physical health	Personal satisfaction
(11, 13, 14)	Lower personal satisfaction is associated with higher engagement in detrimental behaviors (e.g., tobacco use, sedentary lifestyle) and higher incidence of affective disorders (depression, anxiety)	Negative correlation with risky behaviors and mental health	
(14–16)	Women with higher personal satisfaction show better adherence to treatment regimens and self-care practices; lower satisfaction linked to poorer adherence and worsened outcomes	Relationship with chronic illness management	
(21–27)	Higher social support and adaptive coping strategies are associated with greater personal satisfaction despite chronic illness; limited support leads to isolation and dissatisfaction	Socioeconomic, social, and coping influences	
(14, 17)	Sustained personal satisfaction protects against the development and progression of chronic diseases	Longitudinal protective effects	
(22, 28)	Decline in estrogen levels is linked to increased risk of cardiovascular disease, osteoporosis, cognitive impairment, hot flashes, sleep disturbances, and mood swings, all of which can reduce personal satisfaction and health	Hormonal fluctuations and menopause symptoms	Biological changes
(29)	Changes in metabolic rate and body composition influence self-perception and satisfaction with physical appearance	Physiological aging and body composition	
(30)	Declines in fertility and perceived sexual attractiveness affect feminine gender roles, marital satisfaction, and increase susceptibility to psychological disorders	Fertility, sexual attractiveness, and gender roles	
(29, 31)	Interconnected physiological and psychological changes cumulatively contribute to reduced overall life satisfaction and heightened vulnerability to mental health challenges	Overall impact on life satisfaction and mental health	
(32–34)	Higher satisfaction with roles such as wife, mother, paid worker, and caregiver is linked to lower depressive symptoms, better self-esteem, and enhanced psychological well-being, regardless of the number of roles held	Satisfaction with multiple social and family roles	Role functioning
(32)	Women who effectively combine family and employment responsibilities or are satisfied homemakers report higher social-role satisfaction and better overall well-being than women with fragmented or less rewarding role patterns	Successful role integration	
(34–36)	Sustained participation in multiple meaningful roles correlates with better self-rated general health, fewer chronic conditions, fewer functional limitations, and better subjective health in midlife	Long-term engagement in meaningful roles	
(2, 37, 38)	Weak or unfulfilling roles, or unaddressed role-related stressors, are associated with poorer mental health, increased psychological distress, and potential development or worsening of chronic health conditions	Role-related stressors and health outcomes	
(39–41)	Higher levels of psychological distress (anxiety, depression, chronic stress) are associated with lower life satisfaction, reduced sense of purpose, and diminished fulfillment in key life domains	Negative impact on personal satisfaction	Psychological distress
(14, 39–41)	Reduced personal satisfaction mediates the relationship between psychological distress and health outcomes by influencing behavioral (e.g., poor diet, inactivity, disrupted sleep, reduced adherence to medical regimens) and physiological pathways (e.g., hypothalamic-pituitary-adrenal axis hyperactivation, systemic inflammation)	Mediating role of personal satisfaction	
(42)	Midlife women with higher personal satisfaction despite distress are more likely to use adaptive coping strategies, seek social support, and engage in health-promoting behaviors, mitigating negative outcomes	Buffering effect through coping and social support	
(39, 40, 42)	Interventions addressing both psychological distress and personal satisfaction, such as cognitive-behavioral therapy, mindfulness, and social support enhancement, improve overall well-being in midlife women	Implications for interventions	
(25, 26, 43)	Higher perceived social support from family, friends, peers, and significant others is associated with greater personal satisfaction and overall well-being	Positive association with personal satisfaction	Social support
(44, 45)	Well-structured social networks (family, friendships, community engagement) attenuate the negative effects of stress and life challenges, enhancing psychological resilience	Buffering effect against stress	
(45)	Social support moderates the relationship between life stress and life satisfaction, aiding women in managing role transitions and family-related challenges	Moderation of stress and life transitions	
(26)	Social support improves adherence to treatment regimens and enhances overall health outcomes in midlife women with chronic conditions	Impact on chronic disease management	
(25, 26, 44)	Fostering social connectedness and strengthening support systems is a key component of interventions to improve holistic physical, psychological, and social well-being	Implications for interventions	
(46–48)	Problem-focused coping, emotion regulation, and proactive stress management strengthen the protective effects of high personal satisfaction on mental and physical health	Adaptive coping enhances health outcomes	Coping mechanisms
(47, 48)	Strategies such as avoidance, rumination, or substance use attenuate the benefits of personal satisfaction and may exacerbate negative health outcomes	Maladaptive coping undermines well-being	
(46, 47, 49)	Effective coping strategies influence the extent to which personal satisfaction translates into positive health behaviors, adherence to medical recommendations, maintenance of social connections, and overall resilience	Coping moderates the relationship between personal satisfaction and health	
(47, 48)	Therapeutic programs that strengthen adaptive coping (e.g., cognitive-behavioral therapy, mindfulness, problem-solving, emotion regulation) enhance women's ability to manage stress and reinforce the positive impact of personal satisfaction on health	Implications for interventions	
(2, 15, 17)	Cultural and social norms prescribe how women should balance caregiving, professional work, and community involvement; perceived gaps between these expectations and personal capacities can amplify stress and reduce the positive impact of personal satisfaction on health	Role strain due to conflicting societal norms	Societal expectations
(49, 50)	Societal expectations moderate the relationship between personal satisfaction and health outcomes; rigid norms may undermine well-being even in women with high personal satisfaction, whereas supportive or flexible contexts enhance the benefits of satisfaction	Moderating effect on health outcomes	
(50–53)	Life stage transitions—children leaving home, caregiving for aging parents, career plateauing, menopause—interact with societal expectations, influencing stress levels, life satisfaction, and physical and mental health outcomes	Impact during midlife transitions	
(15, 49)	Interventions and policies that promote social awareness, realistic goal-setting, supportive communities, and challenge restrictive gender norms can strengthen the relationship between personal satisfaction and positive health outcomes	Implications for interventions and policies	

#### 3.1.1 Personal satisfaction

Personal satisfaction among women at midlife defined as individual's subjective evaluation of their life and its various domains is positively correlated with adherence to preventive health behaviors, decreased prevalence of chronic diseases, and enhanced neurocognitive performance (11–13). Conversely, diminished personal satisfaction correlates significantly with an increased predilection for detrimental lifestyle choices, including, but not limited to, tobacco use and engagement in sedentary activities; additionally, this demographic exhibits a disproportionately higher incidence of affective disorders such as clinical depression and generalized anxiety disorder (14).

Furthermore, findings indicate that higher levels of personal satisfaction are associated with greater adherence to treatment plans and a more proactive engagement in self-care practices among women managing chronic health conditions. Contrastingly, reduced personal satisfaction is frequently associated with compromised adherence to prescribed therapeutic regimens and a discernible decline in proactive self-care behaviors, potentially culminating in the exacerbation of pre-existing health conditions and a concomitant increase in the incidence of adverse health events. The data suggest that women with higher levels of personal satisfaction are more likely to actively seek information about their health, engage in regular physical activity, maintain a balanced diet, and adhere to recommended screening guidelines (14–16).

Longitudinal studies reveal that sustained personal satisfaction can act as a protective factor against the development and progression of chronic diseases such as cardiovascular disease, diabetes, and certain types of cancer (14, 17). Thus, cultivating personal fulfillment emerges as a pivotal factor in fostering proactive health management, underscoring the intricate relationship between psychological equilibrium and tangible physical health advantages (18, 19). Research indicates that individuals with chronic illnesses often report lower levels of life satisfaction compared to their healthier counterparts. For instance, numerous studies found that people with chronic pain conditions exhibited significantly reduced satisfaction in various life domains, including social interactions and emotional well-being (14, 17, 20, 21). Moreover, the interplay between health and personal satisfaction is influenced by factors such as socioeconomic status, social support, and coping mechanisms. People with large social networks and adaptive coping strategies tend to experience higher levels of personal satisfaction despite managing chronic ailments (21–24). Conversely, those with limited support are more susceptible to feelings of isolation and dissatisfaction (25–27).

#### 3.1.2 Biological changes

During middle age, women experience significant biological changes, primarily driven by hormonal fluctuations, which profoundly impact their physical and mental well-being and, consequently, their personal satisfaction (28). The decline in estrogen levels, for instance, is directly linked to an increased risk of cardiovascular disease, osteoporosis, and cognitive impairment, all of which can significantly diminish perceived personal satisfaction and overall health status. These hormonal shifts also contribute to menopausal symptoms such as hot flashes, sleep disturbances, and mood swings, further complicating the maintenance of personal satisfaction and potentially leading to body-image dissatisfaction (22). Furthermore, the physiological changes associated with aging, including shifts in metabolic rate and body composition, can influence self-perception and satisfaction with one's physical appearance (29). This period of physiological transformation can also engender a decline in fertility and sexual attractiveness, which are often significant to feminine gender roles, potentially reducing marital satisfaction and increasing the risk of psychological disorders (30). The cumulative effect of these interconnected physiological and psychological changes frequently manifests as a decline in overall life satisfaction and an elevated susceptibility to mental health challenges in middle-aged women (31). Therefore, it is imperative that health interventions addressing middle-aged women prioritize strategies that enhance personal satisfaction, recognizing its multifaceted influence on both mental and physical health trajectories (29).

#### 3.1.3 Role functioning

Role functioning refers to how many roles a woman occupies and the quality of her experiences in those roles. Research indicates that higher satisfaction with roles such as wife, mother, paid worker, and caregiver is associated with lower depressive symptoms, independent of the sheer number of roles held (32). Women who successfully combine family and employment responsibilities or who are satisfied homemakers often report the highest social-role satisfaction and better overall well-being compared with women experiencing fragmented or less rewarding role patterns (32). High satisfaction in social and family roles has also been linked to fewer depressive symptoms, higher self-esteem, and better overall psychological well-being in midlife (33, 34). In contrast, weak or unfulfilling role ties are associated with poorer mental health and an increased risk of psychological distress. Long-term engagement in multiple meaningful roles, such as employee, partner, and parent, correlates with better self-rated general health in the mid-50s, and these advantages are not explained solely by earlier health status (35). Greater engagement and satisfaction across roles are also associated with fewer chronic conditions, fewer functional limitations, and better subjective health in midlife. Conversely, women with limited labor-market participation or restricted role patterns exhibit higher rates of obesity and less favorable overall health profiles (34–36).

The relationship between role functioning and personal satisfaction reflects the extent to which a woman perceives herself as effectively fulfilling her occupational, familial, and social responsibilities during midlife (2, 37). Women who experience competence and fulfillment in these roles tend to report higher levels of subjective well-being, indicating that role satisfaction is a key determinant of personal satisfaction. Conversely, unaddressed role-related stressors can exacerbate psychological distress and contribute to the development or worsening of chronic health conditions (38). These stressors affect both mental and physical health, underscoring the interconnectedness of psychological well-being and physiological outcomes.

#### 3.1.4 Psychological distress

Psychological distress, encompassing symptoms of anxiety, depression, and chronic stress, exerts a significant influence on personal satisfaction, which in turn affects health outcomes in women at midlife. Higher levels of distress are consistently associated with reduced personal satisfaction, which manifests as lower life satisfaction, diminished sense of purpose, and reduced fulfillment in key life domains (39–41). Reduced personal satisfaction acts as a mediator between psychological distress and health outcomes by influencing both behavioral and physiological pathways. Women experiencing lower life satisfaction are more likely to engage in maladaptive health behaviors, including poor diet, physical inactivity, disrupted sleep, and reduced adherence to medical regimens, all of which increase susceptibility to chronic conditions such as cardiovascular disease, metabolic syndrome, and weakened immune function (14, 39, 40). Furthermore, diminished personal satisfaction can exacerbate stress-related physiological responses, including hyperactivation of the hypothalamic-pituitary-adrenal (HPA) axis and increased systemic inflammation, thereby linking psychological distress to tangible physical health consequences (40, 41).

Conversely, when midlife women maintain higher personal satisfaction despite experiencing psychological distress, they are more likely to employ effective coping strategies, seek social support, and engage in health-promoting behaviors (42). This buffering effect underscores the critical role of personal satisfaction as mediator, translating reductions in distress into improved mental and physical health outcomes. Recognizing this pathway highlights the importance of interventions that simultaneously address psychological distress and promote personal satisfaction, such as cognitive-behavioral therapy, mindfulness-based programs, and social support enhancement, to optimize overall well-being in midlife women.

#### 3.1.5 Social support

Social support is defined as perceived resources provided by others within an individual's social network, such as family, friends, peers, or significant others, that contribute to emotional, informational, or practical assistance. Enhanced perceived social support is strongly associated with higher personal satisfaction, with multiple studies demonstrating a protective relationship between robust social networks and a range of health outcomes (25, 26, 43). Accordingly, fostering social connectedness and strengthening support systems are critical components of interventions aimed at improving the holistic well-being of midlife women. Well-structured social support networks, including strong familial ties, enduring friendships, and active community engagement, have been consistently shown to buffer the adverse effects of stress and life challenges. Such support not only enhances psychological resilience but also facilitates the adoption of effective coping strategies (44). In a foundational study, Darling et al. (45) observed that midlife women experiencing higher levels of life stress reported lower life satisfaction; however, coping and social support moderated this relationship. This indicates that social support can both attenuate negative health outcomes and promote a more positive appraisal of life, aiding women in navigating role transitions and family-related stress.

The protective effects of social support are particularly evident in the context of chronic disease management, where it has been shown to improve adherence to treatment regimens and enhance overall health outcomes (26). Collectively, these findings underscore the importance of integrating social support enhancement into programs aimed at promoting the physical, psychological, and social well-being of women at midlife.

#### 3.1.6 Coping mechanisms

Coping is a multifaceted construct encompassing the cognitive, behavioral, and emotional strategies individuals use to manage stressful situations (46). In the context of midlife women, coping mechanisms serve as important moderators in the relationship between personal satisfaction and health outcomes. Specifically, the effectiveness of one's coping strategies can influence the extent to which personal satisfaction translates into positive health behaviors and overall well-being. Adaptive coping strategies, such as problem-focused coping, emotion regulation, and proactive stress management, enhance the protective effects of high personal satisfaction on both mental and physical health. In contrast, maladaptive strategies, such as avoidance, rumination, or substance use, can attenuate these benefits, potentially exacerbating negative health outcomes (47, 48).

The proficiency with which midlife women navigate the complex challenges inherent to this developmental stage has a substantial impact on their long-term health trajectories (49). Coping strategies influence not only psychological resilience but also the adoption of health-promoting behaviors, such as regular physical activity, adherence to medical recommendations, and maintenance of social connections, which together moderate the effect of personal satisfaction on health outcomes (47). Accordingly, therapeutic interventions that focus on strengthening adaptive coping mechanisms represent a critical avenue for promoting comprehensive health and enduring well-being in midlife women. Programs incorporating cognitive-behavioral techniques, mindfulness-based stress reduction, problem-solving training, and emotion regulation skills can enhance women's ability to manage stress effectively, thereby reinforcing the positive impact of personal satisfaction on health outcomes (48). By targeting coping as a moderating factor, such interventions optimize the translation of subjective well-being into tangible health benefits.

#### 3.1.7 Societal expectations

Societal expectations play a critical role in shaping women's experiences of personal satisfaction and health outcomes, particularly during midlife (49). Cultural norms and social roles prescribe how women should balance multiple responsibilities, such as caregiving, professional work, and community involvement, which can either support or constrain personal fulfillment. These expectations often amplify stress when women perceive a gap between societal ideals and their own capacities, potentially weakening the positive impact of personal satisfaction on health outcomes. For instance, women who feel pressured to excel simultaneously as employees, mothers, and caregivers may experience role strain, which can undermine the psychological and physiological benefits typically associated with high personal satisfaction (2, 15, 17). Empirical evidence suggests that societal expectations can act as a moderator: the extent to which personal satisfaction translates into improved health outcomes depends on the alignment between women's perceived achievements and prevailing cultural norms. In contexts where societal pressures are high and rigid, even women with high personal satisfaction may experience stress-related health consequences if they perceive themselves as failing to meet external standards. Conversely, in supportive social environments where expectations are flexible or aligned with individual goals, personal satisfaction more effectively promotes adaptive health behaviors, resilience, and well-being (50).

The moderating effect of societal expectations is particularly relevant in midlife, a developmental stage marked by transitions such as children leaving home, caregiving for aging parents, career plateauing, and physiological changes like menopause. Women navigating these transitions within restrictive societal norms may experience diminished life satisfaction and elevated stress, negatively impacting both mental and physical health (50–53). In contrast, women embedded in more egalitarian or supportive cultural contexts can leverage their personal satisfaction to buffer stress and enhance health outcomes. This underscores the importance of considering societal and cultural influences when examining pathways from personal satisfaction to health. Interventions aiming to improve health outcomes in midlife women should therefore account for societal expectations, promoting social awareness, realistic goal-setting, and the development of supportive communities (15, 49). Policies and programs that challenge restrictive gender norms, reduce role overload, and foster autonomy can strengthen the link between personal satisfaction and positive health outcomes, ensuring that subjective well-being more effectively translates into holistic health benefits.

### 3.2 Conceptual concepts

King (9) proposed that, when examining the phenomenon of nursing in relation to individual health, it pertains to the personal system. Accordingly, the middle-range theory developed in this work draws upon the personal system within King's Conceptual System to explain the relationships between personal satisfaction and health outcomes. The dimensions of the personal system pertinent to this phenomenon include perception, growth, and development. Within this framework, personal satisfaction is situated under the dimension of perception, whereas growth and development encompass age-related changes, including biological changes, role functioning, and psychological distress. In this context, the middle-aged woman is viewed as a multidimensional personal system that continuously interacts with internal and external environments in order to maintain health and effectively fulfill social roles. Thus, environmental stressors and health emerge as key concepts from King's Conceptual System incorporated into this theoretical framework.

Environment is defined as internal and external stressors that influence how a person perceives events and interacts with others King (9). King's Conceptual System emphasized the dynamic interactions between individuals, their perceptions, and their environments, offering a holistic perspective that acknowledges the interconnectedness of these factors in shaping health outcomes. In this context, environmental stressors are the age-related stressors that emerge or intensify during the middle-age period arising from normal aging processes, shifting life responsibilities, and changing family, work, and health dynamics. Specifically, age-related stressors incorporates social support, coping mechanisms, and societal expectations.

Health is a dynamic state in the life cycle in which individuals continuously adjust to internal and external stressors to achieve maximum potential for daily functioning King (9). In the proposed theory, health is conceptualized as the outcome of the interaction between the personal system (personal satisfaction and age-related changes) and the environment (age-related stressors). It encompasses health behaviors, physiological responses, and the overall well-being of women at midlife.

This middle-range theory posits that personal satisfaction, defined as an individual's subjective evaluation of their overall contentment and sense of fulfillment across various domains of life, serves as a crucial psychosocial resource that can buffer the negative effects of age-related changes and promote positive health outcomes in women at midlife (54). This middle-range theory considers both the emotional and social contexts that heavily influence the midlife experience.

**Figure 3:**
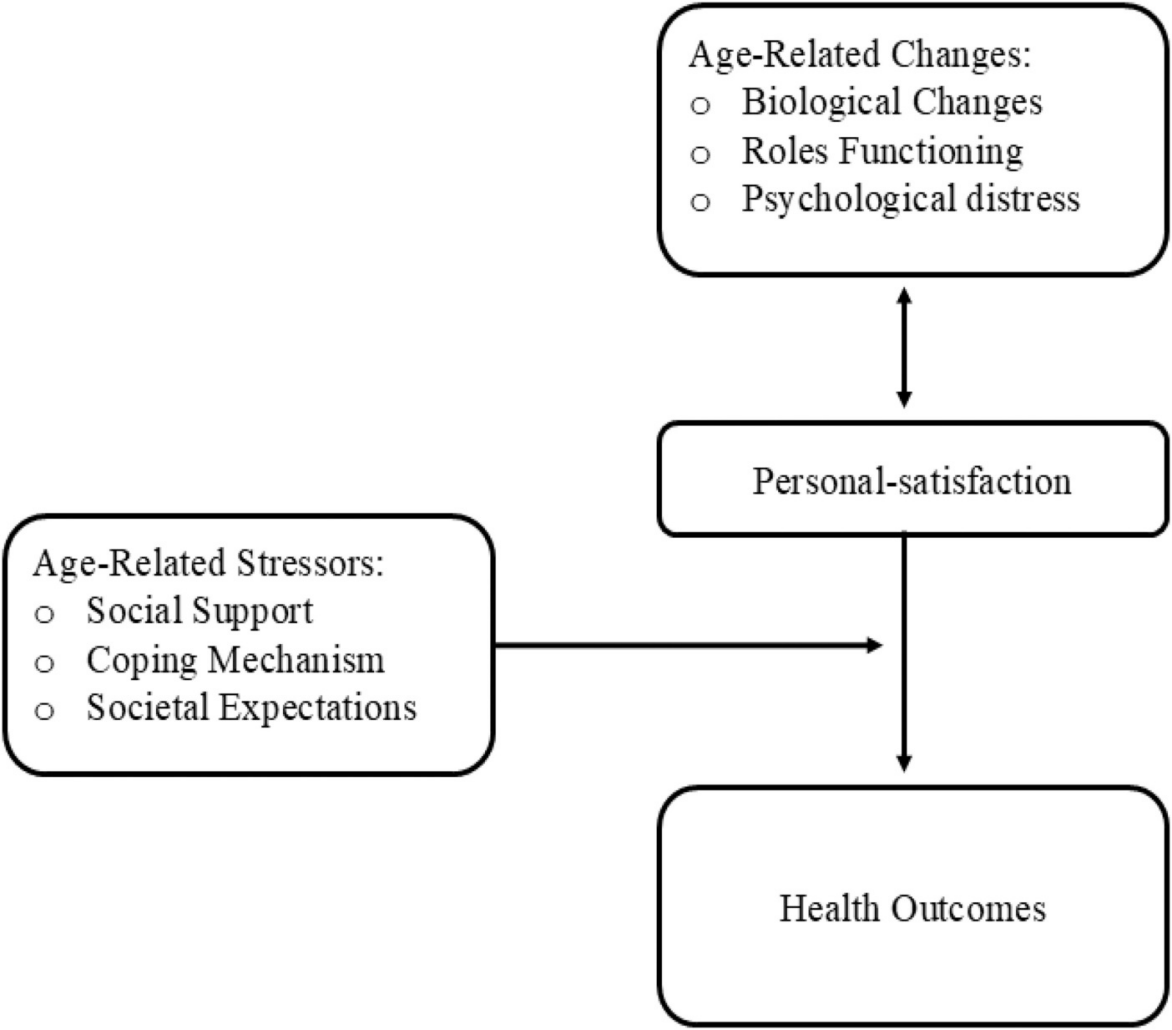
Personal satisfaction and health for women at midlife theory.

The middle-range theory further emphasizes the importance of considering the unique social and cultural contexts in which health experiences of women at midlife are embedded, acknowledging that factors such as socioeconomic status, ethnicity, and access to healthcare resources can significantly shape both their levels of personal satisfaction and their health outcomes (4). The theoretical underpinnings of this middle-range theory are informed by quantitative data obtained from established psychometric instruments designed to evaluate personal satisfaction, age-related changes, age-related stressors, and diverse health outcomes. See Figure 3.

## 4 Discussion

### 4.1 Description of the middle-range theory of personal satisfaction and health for women at midlife

Personal satisfaction, as a construct, encompasses an individual's subjective evaluation of their life and its various domains, reflecting a sense of contentment, fulfillment, and overall well-being. For women at midlife, this construct is particularly relevant, as they often navigate multiple roles and responsibilities, including career, family, caregiving, and personal development, all of which contribute to their overall sense of satisfaction (54). It is crucial to understand the meaning of these events for each woman, rather than focusing solely on the presence or absence of symptoms (55). The experience of women at midlife is influenced by environmental and social contexts (10). There are factors that set apart those women who have a strong sense of well-being in midlife from those who do not (1, 2, 56). A middle-range theory between grand theories and specific empirical findings provides a valuable framework for examining the relationship between personal satisfaction and health of women at midlife (57).

This theoretical approach allows us to bridge the gap between broad conceptualizations of well-being and the specific health challenges and opportunities that women face during this life stage. A failure to adequately address the symptoms and health effects of age-related changes can lead to frustration and dissatisfaction among women seeking healthcare (4). Consequently, therapeutic interventions that cultivate personal satisfaction by strengthening self-identity, enhancing perceived competence in major life roles, and reinforcing supportive social connections may prove instrumental in enhancing the overall well-being of women at midlife navigating the complexities of this developmental stage.

### 4.2 Theoretical concepts

The middle-range theory comprises several key constructs that are interconnected and contribute to a holistic understanding of the relationship between personal satisfaction and health of women at midlife. These constructs include personal satisfaction; age-related changes which encompass biological changes, role functioning, and psychological distress; age-related stressors which encompass, social support, coping mechanisms, and societal expectations; and health outcomes. Table 2 summarizes the key constructs, providing theoretical and empirical definitions alongside their practical empirical applications.

**Table 2:** Definitions of key constructs.

Construct	Theoretical definition	Empirical definition	Empirical application
1. Personal satisfaction	Women's subjective evaluation of their overall contentment and fulfillment across various domains of life.	Personal satisfaction is measured using validated self-report instruments that assess women's overall life satisfaction, and/or satisfaction within specific domains such as body image or self-perception. Higher scores reflect greater levels of personal satisfaction.	The satisfaction with life scale (SWLS) higher scores indicate greater personal satisfaction. Body-image satisfaction scale Rosenberg self-esteem scale. Relationship assessment scale
2. Age-related changes	Age-related changes refer to the interconnected biological, social, and psychological shifts that accompany the transition through midlife.		
2.1. Biological changes	Refer to the physiological transformations that occur during middle adulthood	Biological changes are operationalized as measurable physiological indicators associated with middle adulthood.	Biomarkers such as blood pressure, body mass index (BMI), hormone levels, lipid profiles, and other clinically assessed parameters that reflect age-related transformations in the body.
2.2. Roles functioning	Role functioning refers to the ability to effectively perform and manage the various social, familial, occupational, and personal roles expected during midlife, as well as to adapt to changes in these roles over time.	Roles functioning is measured through validated self-report scales or structured questionnaires that assess women's perceived effectiveness, satisfaction, and balance in managing multiple life roles (e.g., caregiver, professional, partner).	Role functioning scale (RFS). Higher scores indicate greater competence and satisfaction in fulfilling these roles.
2.3. Psychological distress	A state of emotional suffering characterized by symptoms of depression, anxiety, and stress, arising when a woman perceives that environmental demands exceed their coping resources.	Emotional distress is measured using validated psychological assessment tools that quantify symptoms of depression, anxiety, and stress	Depression, anxiety, stress scales (DASS-21). Higher scores indicate greater levels of emotional distress.
3. Age-related stressors	Age-related stressors are the multifactorial pressures associated with navigating midlife transitions. These include the evolving nature of social support systems, the refinement or strain of coping processes, and societal expectations that dictate normative roles and responsibilities.		
3.1. Social support	Refers to the assistance, encouragement, and sense of belonging that women receive from their social relationships, which helps them manage stress, maintain well-being, and achieve personal goals.	Social support is measured through validated self-report questionnaires that assess the perceived availability and quality of help, emotional encouragement, and companionship from family, friends, and significant others. Higher scores indicate greater perceived social support.	The multidimensional scale of perceived social support (MSPSS) The social support questionnaire (SSQ).
3.2. Coping mechanism	Cognitive, emotional, and behavioral strategies that women employ to manage, reduce, or adapt to internal and external stressors.	Coping mechanisms are measured using validated self-report instruments that assess the frequency and type of strategies women use to handle stress, such as problem-focused, emotion-focused, and avoidance-based coping.	The brief COPE The COPE inventory. Higher scores indicate greater use of coping strategies
3.3. Societal expectations	The shared beliefs, values, and standards within a society or culture that prescribe the norms, roles, behaviors, and responsibilities deemed appropriate for middle-aged women.	Societal expectations are measured using validated survey instruments that assess women's perceptions of culturally prescribed roles, norms, and responsibilities expected of them during midlife.	The gender role beliefs scale (GRBS).
4. Health outcomes	Changes in an individual's physical, mental, and social well-being that result from the interplay between personal satisfaction, age-related changes, and age-related stressors.	Health outcomes are measured using a combination of validated self-report scales and objective clinical indicators that assess physical, mental, and social well-being.	The SF-36 for overall health status, The clinical markers (e.g., blood pressure, BMI, cholesterol levels). Psychological assessments for mental health.

#### 4.2.1 Personal satisfaction

Personal satisfaction, defined as an individual's subjective evaluation of their overall contentment and sense of fulfillment across various domains of life, serves as a crucial psychosocial resource that can buffer the negative effects of age-related changes and promote positive health outcomes in women at midlife.

#### 4.2.2 Age-related changes

Age-related changes refer to the interconnected biological, social, and psychological shifts that accompany the transition through midlife. These changes significantly influence health outcomes and can both impact and be influenced by personal satisfaction.

##### 4.2.2.1 Biological changes

Biological changes defines as the physiological transformations that occur during middle adulthood.

##### 4.2.2.2 Role functioning

Role functioning refers to the ability to effectively perform and manage the various social, familial, occupational, and personal roles expected during midlife, as well as to adapt to changes in these roles over time.

##### 4.2.2.3 Psychological distress

Psychological distress is a state of emotional suffering characterized by symptoms such as anxiety, depression, irritability, and feelings of overwhelm, often arising from perceived challenges, stressors, or life transitions. It reflects a disruption in an individual's emotional and cognitive balance, which can negatively affect functioning and overall well-being.

#### 4.2.3 Age-related stressors

Age-related stressors are the multifactorial pressures associated with navigating midlife transitions. These include the evolving nature of social support systems, the refinement or strain of coping processes, and societal expectations that dictate normative roles and responsibilities.

##### 4.2.3.1 Social support

Social support refers to the assistance, encouragement, and sense of belonging that women receive from their social relationships, which helps them manage stress, maintain well-being, and achieve personal goals.

##### 4.2.3.2 Coping mechanisms

Coping mechanisms are the cognitive and behavioral strategies that individuals use to manage, reduce, or tolerate internal and external stressors. These strategies help maintain psychological equilibrium, promote problem-solving, and support emotional well-being in the face of challenges, life transitions, or adverse circumstances.

##### 4.2.3.3 Societal expectations

Societal expectations refer to the norms, roles, behaviors, and responsibilities that a culture or society prescribes for individuals based on factors such as age, gender, social status, or life stage. These expectations influence how individuals perceive themselves, make decisions, and fulfill personal and social roles, often shaping stress levels, behavior, and overall well-being.

#### 4.2.4 Health outcomes

The ultimate outcome of the interplay between personal satisfaction, age-related changes, and age-related stressors is reflected in various health outcomes, including physical health, mental health, and health-related behaviors.

### 4.3 Assumptions

The middle-range theory rests on several key assumptions that underpin its propositions and provide a framework for understanding the relationship between personal satisfaction and health of women at midlife. First, personal satisfaction is a multifaceted construct that comprises an individual's subjective evaluation of their overall contentment and fulfillment in life, encompassing various life domains, such as relationships, career, personal growth, and leisure activities. Secondly, women at midlife encounter distinctive health-related challenges and transitions that can significantly influence their well-being, including, but not limited to, hormonal fluctuations, the onset of chronic conditions, and age-related physical changes. Third, health is acknowledged as a multifaceted construct, transcending the mere absence of disease, and encompassing a holistic spectrum of physical, psychological, and social dimensions that intricately interweave to shape an individual's overall state of well-being. Lastly, strategically designed interventions that target enhancements in personal satisfaction have the potential to elicit beneficial outcomes concerning the overall health status of women at midlife, contingent upon their careful adaptation to meet the distinct needs and specific circumstances inherent to this particular population cohort.

### 4.4 Propositions

The following propositions, derived from the theory, offer specific, testable statements regarding the relationships among the concepts.

Proposition 1: Personal satisfaction acts as a mediator between age-related changes and health outcomes, where higher personal satisfaction mitigates the negative effects of age-related changes on health.

Proposition 2: Elevated levels of personal satisfaction are positively correlated with enhanced health outcomes among women at midlife, reflecting a synergistic interplay between subjective well-being and objective health indicators.

Proposition 3: Biological changes, role functioning, and psychological distress influence personal satisfaction in midlife women. In particular, biological changes and psychological distress can reduce women's personal satisfaction, while the extent to which women perceive competence and fulfillment in their multiple roles positively affects their personal satisfaction.

Proposition 4: Social support, coping mechanisms, and societal expectations moderate the association between personal satisfaction and health outcomes in women at midlife. Women who perceive higher levels of social support, utilize more adaptive coping strategies, and experience more favorable societal expectations demonstrate greater personal satisfaction and improved health outcomes.

### 4.5 Hypotheses

The proposed theory will be used with women at midlife to test the following hypotheses:

#### 4.5.1 Hypothesis 1

Higher levels of personal satisfaction weaken the negative impact of age-related stressors on middle-aged women's health.

#### 4.5.2 Hypothesis 2

Women at midlife reporting higher levels of personal satisfaction will exhibit superior physical health outcomes, including lower rates of chronic diseases and improved physiological functioning.

#### 4.5.3 Hypothesis 3

Women at midlife reporting higher levels of personal satisfaction will demonstrate enhanced mental health outcomes, including reduced prevalence of depression, anxiety, and other psychological disorders.

#### 4.5.4 Hypothesis 4

Biological changes and psychological distress are negatively associated with personal satisfaction, while higher perceived role functioning is positively associated with personal satisfaction among midlife women.

#### 4.5.5 Hypothesis 5

The positive association between personal satisfaction and health outcomes will be more pronounced among women at midlife who perceive higher levels of social support, employ more adaptive coping strategies, and experience more favorable societal expectations.

## 5 Implications for nursing practice

This middle-range theory underscores the essential role of nurses in adopting a holistic approach to patient care. Nurses can integrate assessments of personal satisfaction into routine health evaluations, utilizing validated tools to gauge patients’ levels of fulfillment, purpose, and overall contentment with their lives. This comprehensive outline provides a holistic blueprint for the delivery of healthcare to women at midlife that incorporates psychological and emotional well-being. The integrative and patient-centered approach improves current healthcare strategies, which focus purely on physical health needs, by acknowledging the impact of age-related changes and stressors.

Furthermore, nurses are strategically positioned to provide counseling and support to women at midlife, empowering them to identify sources of personal satisfaction and implement strategies for enhancing their overall well-being. This may involve facilitating access to resources and programs that promote social connectedness, life-long learning, and engagement in meaningful activities tailored to their individual interests and preferences.

## 6 Limitations

Despite the comprehensive insights offered by this middle-range theory, several limitations must be acknowledged. A significant concern is the predominance of cross-sectional designs in many supporting studies, which limits the ability to establish causal relationships or observe changes in personal satisfaction and health outcomes over time. Additionally, much of the existing research relies on self-reported measures of personal satisfaction and health, introducing potential biases such as social desirability or recall errors. This reliance on subjective data underscores the need for future studies to incorporate objective indicators, including physiological markers, clinical assessments, and longitudinal tracking, to strengthen the validity and generalizability of findings. Moreover, the current literature often underrepresents diverse populations and contextual factors that may moderate the relationships between age-related changes, satisfaction, and health, suggesting that future research should adopt more inclusive and culturally sensitive approaches to better capture the complexity of these dynamics.

## 7 Directions for future research

Future investigations should employ longitudinal study designs to capture the dynamic interplay between personal satisfaction and health outcomes over extended periods, enabling a more comprehensive understanding of causal relationships and potential feedback loops, while paying attention to confounding factors, such as socioeconomic status, cultural contexts, and access to healthcare resources, which may further refine the understanding of the relationship between personal satisfaction and women's health during midlife (1, 3, 4).

Future studies should explore the effectiveness of various intervention strategies aimed at enhancing personal satisfaction among women at midlife, encompassing both individual-level approaches (e.g., cognitive-behavioral therapy, mindfulness-based interventions) and systemic-level interventions designed to address broader socio-cultural determinants of health and well-being, which will aid in identifying specific strategies that demonstrate the greatest potential for improving women's health outcomes across diverse populations and settings. To enhance the generalizability of future research, it is imperative to include heterogeneous populations, encompassing women from diverse socioeconomic strata, cultural backgrounds, geographical locations, and ethnic origins, allowing for a more nuanced understanding of how personal satisfaction influences health across various demographic subgroups. Additionally, qualitative research methods may prove valuable in elucidating the lived experiences of women at midlife, capturing the richness and complexity of their perceptions, values, and priorities, ultimately informing the development of more culturally sensitive and contextually relevant interventions.

Recognizing the intricate network of factors that shape the well-being of women during midlife necessitates a multifaceted approach that actively challenges and transcends the conventional stereotypes and implicit biases that have historically permeated healthcare practices and research paradigms, thereby promoting a more equitable and comprehensive understanding of women's health experiences (58–60).

## 8 Conclusion

In conclusion, this study offers a comprehensive investigation into the intricate relationships between personal satisfaction and health among women at midlife, providing a nuanced framework for understanding the multiple pathways through which personal satisfaction influences overall health during this critical life stage. The results underscore the importance of adopting a holistic, person-centered approach to healthcare that recognizes the interconnectedness of mind, body, and social context in promoting women's health and well-being throughout the lifespan. These findings contribute valuable insights to the development of targeted interventions and policies designed to empower women to cultivate personal satisfaction, enhance their health, and thrive during their midlife and beyond.

## Data Availability

The original contributions presented in the study are included in the article/Supplementary Material, further inquiries can be directed to the corresponding author.

## References

[1] Melinda U Phang K Abdullah KL Ooi PB. Midlife progression and beyond: a systematic review of middle-aged women’s perspectives and experiences. Educ Gerontol. (2024) 50(5):367–85. 10.1080/03601277.2023.2293580

[2] Thomas AJ Mitchell ES Woods NF. The challenges of midlife women: themes from the Seattle midlife women’s health study. Women’s Midlife Health. (2018) 4(8):1–10. 10.1186/s40695-018-0039-9 30766718 PMC6298022

[3] Alspaugh A Im E-O Reibel MD Barroso J. The reproductive health priorities, concerns, and needs of women in midlife: a feminist poststructuralist qualitative analysis. Qual Health Res. (2021) 31(4):1049732320970491. 10.1177/1049732320970491 33213259

[4] King K Soylemez K Lusher J. The lived experience of menopause and journeying to the other side. World J Adv Res R. (2024) 24:580–90. 10.30574/wjarr.2024.24.2.3338

[5] Karakcheyeva V Willis-Johnson H Corr PG Frame LA. The well-being of women in healthcare professions: a comprehensive review [review of the well-being of women in healthcare professions: a comprehensive review]. Glob Adv Integr Med Health. (2024) 13:1–10. 10.1177/27536130241232929 PMC10859069 38344248

[6] Peters MD Marnie C Tricco AC Pollock D Munn Z Alexander L Updated methodological guidance for the conduct of scoping reviews. JBI evidence Synthesis. (2020) 18(10):2119–26. 10.11124/JBIES-20-00167 33038124

[7] Fawcett J DeSanto-Madeya S. Contemporary Nursing Knowledge: Analysis and Evaluation of Nursing Models and Theories. 3rd ed. Philadelphia: F.A. Davis Co (2012).

[8] King I. King’s conceptual system, theory of goal attainment, and transaction process in the 21st century. Nurs Sci Q. (2007) 20(2):109–11. 10.1177/0894318407299846 17447333

[9] King I. A systems approach in nursing administration: structure process and outcome. Nurs Adm Q. (2006) 30(2):100. 10.1097/00006216-200604000-00006 16648721

[10] King I. Health as the goal for nursing. Nurs Sci Q. (1990) 3(3):123. 10.1177/089431849000300307 2392262

[11] Ashgar RI Thilagavathi K. Health promotion behaviors and psychosocial factors among middle-aged women in Saudi Arabia. SAGE Open Nursing. (2023) 9:1–11. 10.1177/23779608231187263 PMC10336765 37448970

[12] Ashgar R. Relationships between personal satisfaction, cardiovascular disease risk, and health promoting behavior among arab American middle-aged women. J Cardiovasc Nurs. (2020) 36(3):273. 10.1097/jcn.0000000000000690 32398497

[13] Edwards ES Sackett SC. Psychosocial variables related to why women are less active than men and related health implications [review of psychosocial variables related to why women are less active than men and related health implications]. Clin Med Insights Womens Health. (2016) 9s1:47–56. 10.4137/cmwh.s34668 PMC4933535 27398045

[14] Kim ES Delaney S Tay L Chen Y Diener E VanderWeele TJ. Life satisfaction and subsequent physical, behavioral, and psychosocial health in older adults. Milbank Quarterly. (2021) 99(1):209. 10.1111/1468-0009.12497 33528047 PMC7984669

[15] Papi S Cheraghi M. Multiple factors associated with life satisfaction in older adults. Menopausal Rev. (2021) 20(2):65. 10.5114/pm.2021.107025 PMC8297631 34321983

[16] Tran T-V-T Franck J-E Cœuret-Pellicer M Rigal L Ringa V Menvielle G. Combined effect of health status and primary care use on participation in cancer screening: the CONSTANCES cohort. Womens Health Rep. (2020) 1(1):511. 10.1089/whr.2020.0096 PMC9380874 35982989

[17] Lukkala PS Honkanen R Rauma PH Williams LJ Quirk SE Kröger H Life satisfaction and morbidity among postmenopausal women. PLoS One. (2016) 11(1):1–10. 10.1371/journal.pone.0147521 PMC4723073 26799838

[18] Amiri M Raei M Sadeghi E Keikavoosi-Arani L Khosravi A. Health-promoting lifestyle and its determining factors among students of public and private universities in Iran. J Educ Health Promot. (2023) 12(1):1–7. 10.4103/jehp.jehp_963_22 37727403 PMC10506751

[19] Kar N. Happiness and health: an intricate relationship. In: Chetri S Dutta T Mandal MK Patnaik P, editors. Understanding Happiness: An Explorative View. Singapore: Springer (2023). p. 205–31. 10.1007/978-981-99-3493-5_9

[20] Cardin F Ambrosio F Amodio P Minazzato L Bombonato G Schiff S Quality of life and depression in a cohort of female patients with chronic disease. BMC Surg. (2012) 12:1–5. 10.1186/1471-2482-12-s1-s10 23173648 PMC3499261

[21] Dong HJ Larsson B Dragioti E Bernfort L Levin LÅ Gerdle B. Factors associated with life satisfaction in older adults with chronic pain (PainS65+). J Pain Res. (2020) 13:475–89. 10.2147/JPR.S234565 32184652 PMC7062502

[22] Ashgar RI. Body image satisfaction as a psychological reaction to age-related developmental changes among middle-aged women in Saudi Arabia. In Proceedings of the Second International Nursing Conference “Nursing Profession in the Current Era” (INC 2023); Atlantis Press (2023). p. 35. 10.2991/978-94-6463-248-4_5

[23] Mohamed A Isa HM. Health related quality of life in patients with chronic diseases. IJMEDPH. (2020) 10(3):104–9. 10.5530/ijmedph.2020.3.22

[24] Wilder LV Pype P Mertens F Rammant E Clays E Devleesschauwer B Living with a chronic disease: insights from patients with a low socioeconomic status. BMC Fam Pract. (2021) 22(1):1–11. 10.1186/s12875-021-01578-7 34789153 PMC8598397

[25] Åslund C Larm P Starrin B Nilsson KW. The buffering effect of tangible social support on financial stress: influence on psychological well-being and psychosomatic symptoms in a large sample of the adult general population. Int J Equity Health. (2014) 13(1):1–9. 10.1186/s12939-014-0085-3 25260355 PMC4189745

[26] Luo Z Zhong S Zheng S Li Y Guan Y Xu W Influence of social support on subjective well-being of patients with chronic diseases in China: chain-mediating effect of self-efficacy and perceived stress. Front Public Health. (2023) 11:1–9. 10.3389/fpubh.2023.1184711 PMC10325675 37427286

[27] Zhou M Lin W. Adaptability and life satisfaction: the moderating role of social support. Front Psychol. (2016) 7:1–7. 10.3389/fpsyg.2016.01134 27516753 PMC4963457

[28] Xie W Zhou J Yu H Cai Z. A study on the relationship between life satisfaction and physical health of retired female athletes. Appl Math Nonli Sci. (2024) 9(1):1–15. 10.2478/amns-2024-1500

[29] Gondek D Bernardi L McElroy E Comolli CL. Why do middle-aged adults report worse mental health and wellbeing than younger adults? An exploratory network analysis of the Swiss household panel data. Appl Res Qual Life. (2024) 19(4):1459. 10.1007/s11482-024-10274-4 39211006 PMC11349807

[30] Khakkar M Kazemi A. Relationship between mental health and climacteric adjustment in middle aged women: a confirmatory analysis. BMC Women s Health. (2023) 23(1):1–7. 10.1186/s12905-023-02397-x 37149575 PMC10164310

[31] Yao J Chen J. A study on the characteristics of middle-aged Chinese female users based on clothing needs. Asian Soc Sci. (2023) 19(4):86. 10.5539/ass.v19n4p86

[32] Perrig-Chiello P Hutchison S Hoepflinger F. Role involvement and well-being in middle-aged women. Women Health. (2008) 48(3):303–23. 10.1080/03630240802463517 19064464

[33] Bień A Niewiadomska I Korżyńska-Piętas M Rzońca E Zarajczyk M Pięta B General self-efficacy as a moderator between severity of menopausal symptoms and satisfaction with life in menopausal women. Front Public Health. (2024) 12:1426191. 10.3389/fpubh.2024.1426191 39267631 PMC11390549

[34] McMunn A Bartley M Hardy R Kuh D. Life course social roles and women’s health in mid-life: causation or selection? J Epidemiol Community Health. (2006) 60(6):484–9. 10.1136/jech.2005.042473 16698977 PMC2563934

[35] Ahrens CJC Ryff CD. Multiple roles and well-being: sociodemographic and psychological moderators. Sex Roles. (2006) 55:801–15. 10.1007/s11199-006-9134-8

[36] Avis NE Colvin A Hess R Bromberger JT. Midlife factors related to psychological well-being at an older age: study of women’s health across the nation. J Womens Health. (2021) 30(3):332–40. 10.1089/jwh.2020.8479 PMC7957375 33090934

[37] Talley AE Kocum L Schlegel RJ Molix L Bettencourt BA. Social roles, basic need satisfaction, and psychological health: the central role of competence. Pers Soc Psychol Bull. (2012) 38(2):155–73. 10.1177/0146167211432762 22215698 PMC4756393

[38] Rindner L Nordeman L Strömme G Svenningsson I Premberg Å Hange D Prognostic factors for future mental, physical and urogenital health and work ability in women, 45–55 years: a six-year prospective longitudinal cohort study. BMC Womens Health. (2020) 20(1):1–10. 10.1186/s12905-020-01015-4 32787825 PMC7425146

[39] Kim S. Factors influencing life satisfaction in middle-aged women in South Korea: a descriptive research using panel data. Nurs Pract Today. (2021) 8(4):265–72. 10.18502/npt.v8i4.6702

[40] Oh J Kim S. The relationship between psychological distress, depressive symptoms, emotional eating behaviors and the health-related quality of life of middle-aged Korean females: a serial mediation model. BMC Nurs. (2023) 22(1):1–9. 10.1186/s12912-023-01303-y 37087430 PMC10122802

[41] Garaigordobil M. Predictor variables of happiness and its connection with risk and protective factors for health. Front Psychol. (2015) 6:1–10. 10.3389/fpsyg.2015.01176 26321990 PMC4532923

[42] Sheikh MM Qayyum R Panda M. Relationship of physicians’ rapport with patients’ satisfaction and psychological well-being during hospitalization. Cureus. (2019) 11(6):e4991. 10.7759/cureus.4991 31497422 PMC6707819

[43] Nazari S Afshar PF Moghdada LS Shabestari AN Farhadi A Sadeghi NK. Association between perceived social support and mental health status among older adults. J Holistic Nurs Midwifery. (2021) 31(3):147. 10.32598/jhnm.31.3.2063

[44] Aivalioti I Pezirkianidis C. The role of family resilience on parental well-being and resilience levels. Psychology. (2020) 11(11):1705. 10.4236/psych.2020.1111108

[45] Darling CA Coccia C Senatore N. Women in midlife: stress, health and life satisfaction. Stress Health. (2012) 28(1):31–40. 10.1002/smi.1398 22259156

[46] Skapinakis P Bellos S Oikonomou A Dimitriadis G Gkikas P Perdikari E Depression and its relationship with coping strategies and illness perceptions during the COVID-19 lockdown in Greece: a cross-sectional survey of the population. Depress Res Treat. (2020) 2020:3158954. 10.1155/2020/3158954 32908697 PMC7450302

[47] Holton MK Barry AE Chaney JD. Employee stress management: an examination of adaptive and maladaptive coping strategies on employee health. Work. (2015) 53(2):299. 10.3233/wor-152145 26409388

[48] Zimmer-Gembeck MJ Skinner EA Modecki KL Webb HJ Gardner AA Hawes T The self-perception of flexible coping with stress: a new measure and relations with emotional adjustment. Cogent Psychology. (2018) 5(1):1537908. 10.1080/23311908.2018.1537908

[49] Park S Yeom HY Sok S. Effects of health promoting education program for Korean middle-aged women. J Phys Ther Sci. (2019) 31(1):5. 10.1589/jpts.31.5 30774196 PMC6348176

[50] Kamyab F Aliasghar H. The psychological impact of social expectations on women’s personal choices. Psychol Woman J. (2023) 4(2):169. 10.61838/kman.pwj.4.2.20

[51] Behere PB Sinha AA Chowdhury D Behere AP Yadav R Nagdive A Woman mental health - midlife. J Pharm Res Int. (2021) 33(37A):69–76. 10.9734/jpri/2021/v33i37a31981

[52] Chawla S Sharma RR. Enhancing women’s well-being: the role of psychological capital and perceived gender equity, with social support as a moderator and commitment as a mediator. Front Psychol. (2019) 10:1–15. 10.3389/fpsyg.2019.01377 31275203 PMC6593049

[53] Maclean H Glynn K Ansara D. Multiple roles and women’s mental health in Canada. BMC Womens Health. (2004) 4:1–9. 10.1186/1472-6874-4-s1-s3 15345066 PMC2096667

[54] Ashgar RI. Personal satisfaction: a concept analysis. Nurs Forum. (2022) 57(3):446. 10.1111/nuf.12692 35005791

[55] King I. A Theory of Nursing: Systems, Concepts, Process. New York: Wiley (1981).

[56] Olchowska-Kotala A. Psychological resources and self-rated health status on fifty-year-old women. J Menopausal Med. (2015) 21(3):133. 10.6118/jmm.2015.21.3.133 26793678 PMC4719087

[57] Liehr P Smith MJ. Middle range theory: a perspective on development and use. ANS Adv Nurs Sci. (2017) 40(1):51–63. 10.1097/ANS.0000000000000162 27930396

[58] Hankivsky O Reid C Cormier R Varcoe C Clark N Benoit C Exploring the promises of intersectionality for advancing women’s health research. Int J Equity Health. (2010) 9(1):5. 10.1186/1475-9276-9-5 20181225 PMC2830995

[59] Soares JM Lópes RD Sorpreso ICE Baracat EC. Women health: holistic view. Revista da Associação Médica Brasileira. (2023) 69:1–3. 10.1590/1806-9282.2023s127 37556646 PMC10411701

[60] Short SE Zacher M. Women’s health: population patterns and social determinants. Annu Rev Sociol. (2022) 48:277–98. 10.1146/annurev-soc-030320-034200 38765764 PMC11101199

